# Identification of a novel scaffold for a small molecule GPR139 receptor agonist

**DOI:** 10.1038/s41598-019-40085-9

**Published:** 2019-03-07

**Authors:** Anne Cathrine Nøhr, Mohamed A. Shehata, Daniel Palmer, Rina Pokhrel, Maria Vallianou, Simon R. Foster, Patrick R. Gentry, David E. Gloriam, Hans Bräuner-Osborne

**Affiliations:** Department of Drug Design and Pharmacology, Faculty of Health and Medical Sciences, University of Copenhagen, Universitetsparken 2, 2100 Copenhagen, Denmark

## Abstract

GPR139 is an orphan G protein-coupled receptor (GPCR) that is primarily expressed in the brain in regions known to regulate motor control and metabolism. Here, we screened a diverse 4,000 compound library in order to identify GPR139 agonists. We identified 11 initial hits in a calcium mobilization screen, including one compound, AC4, which contains a different chemical scaffold to what has previously been described for GPR139 agonists. Our mutagenesis data shows that AC4 interacts with the same hotspots in the binding site of GPR139 as those reported to interact with the reference agonists 1a and 7c. We additionally tested and validated 160 analogs in a calcium mobilization assay and found 5 compounds with improved potency compared to AC4. In total, we identified 36 GPR139 agonists with potencies in the nanomolar range (90–990 nM). The most potent compounds were confirmed as GPR139 agonists using an orthogonal ERK phosphorylation assay where they displayed a similar rank order of potency. Accordingly, we herein introduce multiple novel GPR139 agonists, including one with a novel chemical scaffold, which can be used as tools for future pharmacological and medicinal chemistry exploration of GPR139.

## Introduction

GPR139 is an orphan class A G protein-coupled receptor (GPCR) originally identified using bioinformatic searches of the human genome^1^. In mammals, GPR139 is predominantly expressed in the central nervous system, with the highest expression observed in the striatum, pituitary, habenula, thalamus and hypothalamus^2–6^. Given its expression profile, GPR139 has been suggested as a therapeutic target for metabolic syndromes^6–10^ or motor diseases^5,9,11^. Despite its putative value as a drug target, GPR139 has proven challenging to study due to the relative paucity of probe compounds with which to characterize the receptor.

**Figure 1:**
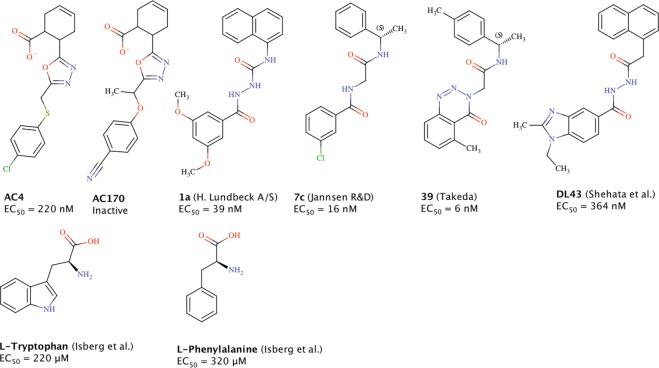
Chemical structures and EC_50_ values of GPR139 agonist compounds: **AC4** and **AC170 (this study)**, **1a**^11^, **7c**^10^, **39**^12^, **DL43**^13^, and L-tryptophan^7^.

Several endogenous GPR139 ligands have been proposed, including the aromatic amino acids L-tryptophan and L-phenylalanine, the adrenocorticotropic hormone (ACTH), and α- and β-melanocyte-stimulating hormone (EC_50_ = 220 μM, 320 μM, 2.1 μM, 2.2 μM, and 6.3 μM, respectively)^5,7,8^. In addition, multiple surrogate GPR139 agonists have been described by Hu *et al*.^9^, Shi *et al*. from Lundbeck A/S^11^, Dvorak *et al*. from Janssen R&D^10^, Hitchcock *et al*. from Takeda Pharmaceutical Company Limited^12^, as well as by Shehata *et al*.^13^ (Fig. 1). In contrast, only few GPR139 antagonists, of which none are potent and selective, have been identified to date^9,14,15^.

Here, we generated a focused compound library, designed to maximize structural diversity while selecting for sensible physicochemical properties, and screened it against GPR139 using a protocol designed to identify both agonist and antagonist hits. We identified 36 novel agonists, including **AC4**, which exhibits unique structural elements unseen in our recently published pharmacophore based on reported GPR139 ligands^13^. Moreover, we utilized site-directed mutagenesis to demonstrate that **AC4** utilizes the same critical residues for binding at GPR139 as previously reported agonists **1a** and **7c**. By screening additional analogs, we identified 5 compounds with improved agonist potency that were confirmed in an orthogonal ERK-phosphorylation assay. Together, these ligands represent promising tool compounds and further expand the variety of chemical structures available with which to characterize GPR139 and to guide future campaigns in the development of surrogate ligands.

## Results

### Identification of novel GPR139 agonists

**Figure 2:**
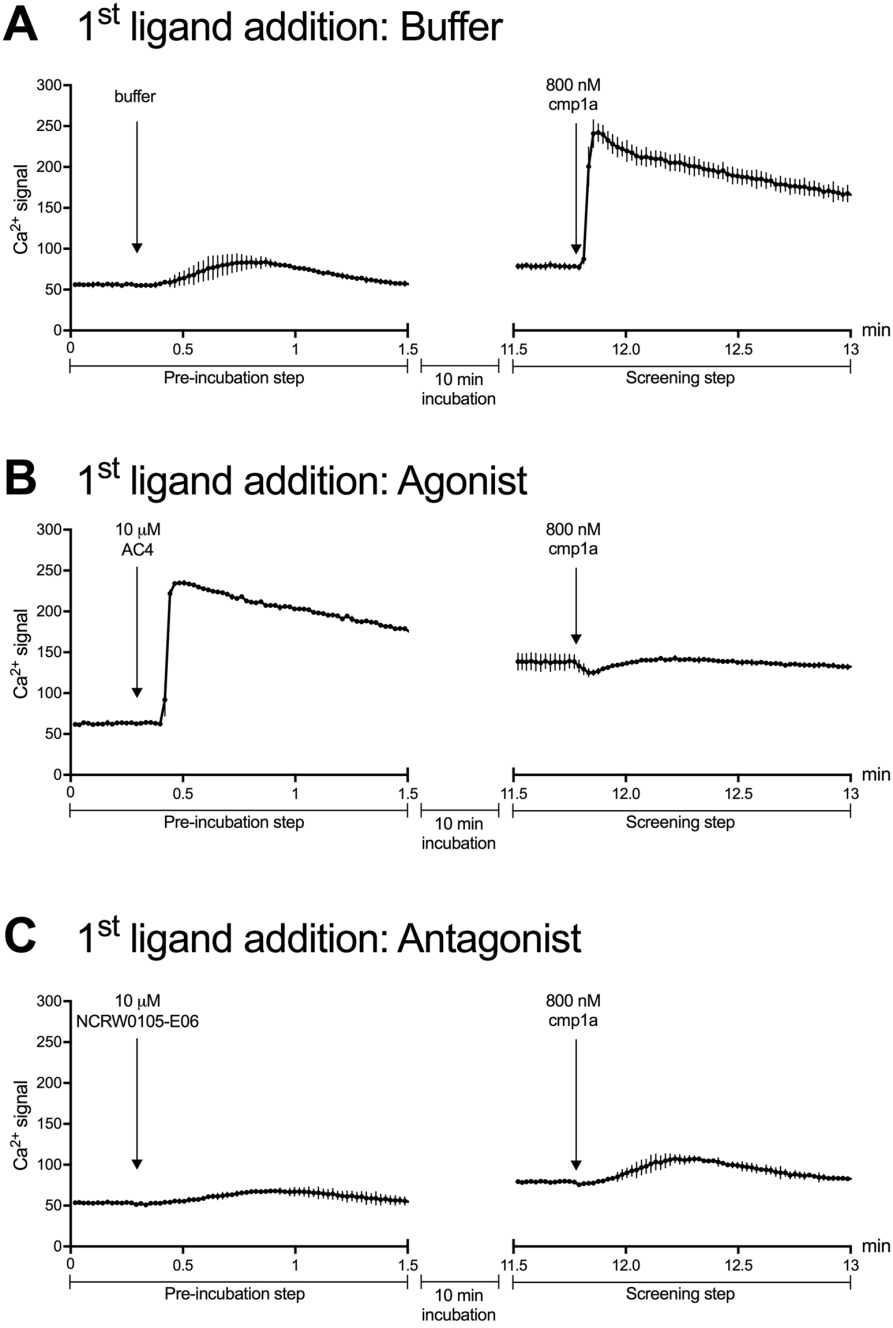
Representative data of the 2-stage screening protocol which enabled simultaneously screening for agonist and antagonists hits by eliciting different response profiles during the initial addition of (**A**) buffer, (**B**) agonist or (**C**) antagonist and subsequent addition of the reference agonist compounds **1a** (cmp1a). Only the second addition of **1a** was measured during the screening campaign and the agonist/antagonist activity was then uncovered during subsequent detailed characterization of the hits.

**Figure 3:**
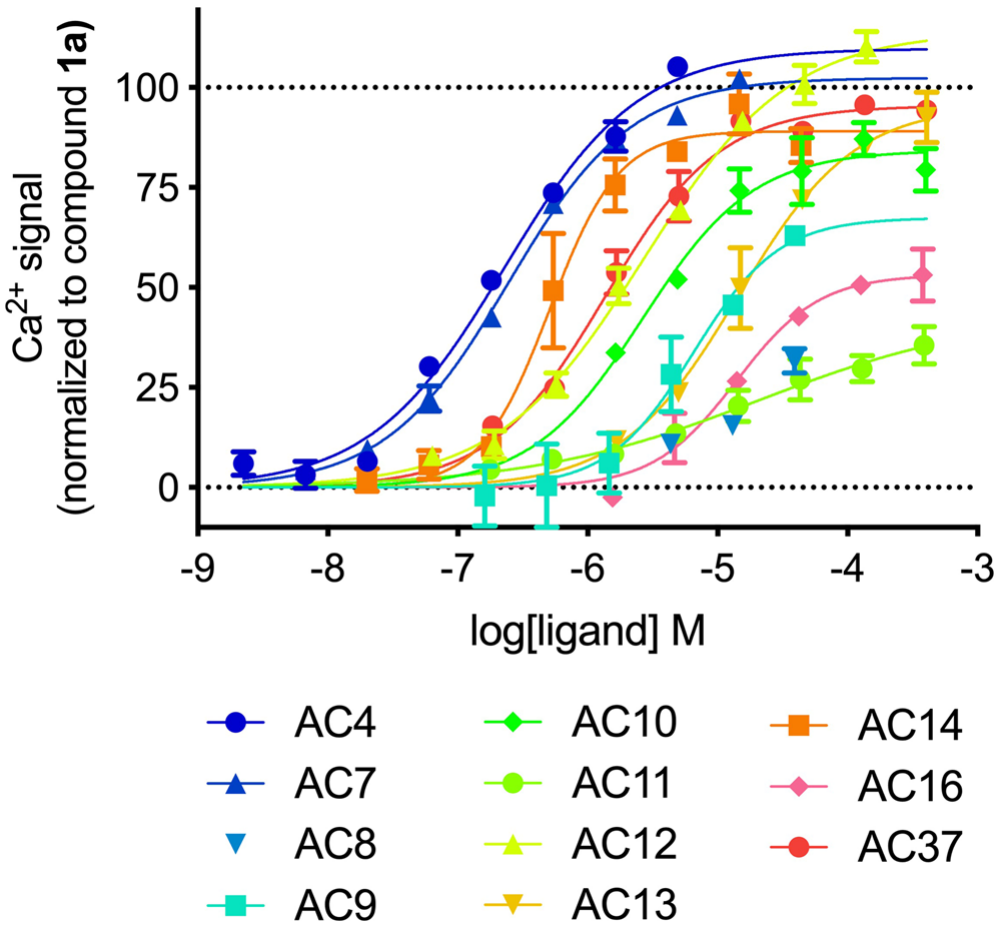
Calcium mobilization concentration-response curves for 11 initial agonist hits from diverse library in CHO-GPR139 cells. assay. The graphs are mean ± S.D. of representative concentration-response curves out of at least three independent experiments performed in duplicate. Data are normalized to the Ca^2+^ response of buffer (0%) and 8 μM **1a** (100%).

To identify novel GPR139 ligand scaffolds, we performed a screening campaign of 4,000 chemically diverse screening compounds in CHO cells stably expressing the GPR139 receptor using a fluorescence-based calcium mobilization assay. Our 2-stage assay protocol enabled us to screen for agonist and antagonists simultaneously. This involved an initial addition of test compounds followed by a second stimulation with an EC_80_ concentration (800 nM) of the reference GPR139 agonist compound **1a**. In this assay format, the addition of agonists led to a Ca^2+^ response during the initial 10 minute recording, although upon stimulation **1a** was not able to activate GPR139 further, due to receptor desensitization in the constant presence of the test agonist (Fig. 2B, example with **AC4** an agonist found in this screening campaign). Initial addition of antagonists did not elicit a Ca^2+^ response during the first compound addition step, but inhibited the response from **1a** in the screening step (Fig. 2C, example shown with a published antagonist NCRW0105-E06)^14^. By way of comparison, the addition of **1a** produced a full Ca^2+^ response in the wells that were initially tested with buffer or with compounds that had no effect on GPR139 (Fig. 2A, example with buffer). In total 11 hits, all agonists, were identified in the primary screen (Figs 3, S1 and Table S1).

### AC4 binds in the same binding site as the known reference agonists

**Figure 4:**
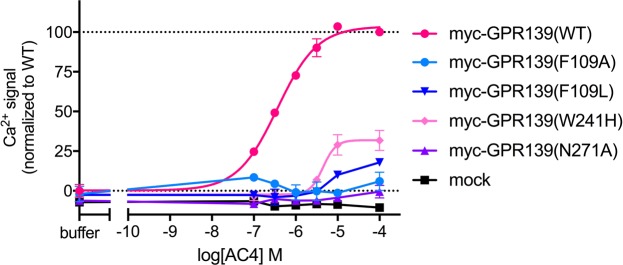
Ca^2+^ mobilization concentration-response curves of **AC4** on GPR139 wild-type (WT) and mutants. Data is shown from a representative experiment (mean ± S.D.) out of three independent experiments performed in duplicate. Data is normalized to the Ca^2+^ response of buffer (0%) and 100 μM **AC4** (100%) on myc-GPR139(WT). Residue numbering in superscript is according to GPCRdb numbering system.

Our recent mutational study of GPR139^16^ indicates that residues F109^3×33^, W241^6×48^ and N271^7×38^ are required for its activation by the two agonists **1a**^11^ and **7c**^10^. In order to assess if the same residues were important for the recognition of **AC4**, the most potent agonist discovered in the primary screen (EC_50_ = 0.22 μM), we tested the ligand on our GPR139 mutants: F109A^3×33^, F109L^3×33^, W241H^6×48^, and N271A^7×38^. The expression levels of F109A^3×33^, F109L^3×33^ and W41H^6×48^ were not significantly different from the N-terminally myc-tagged GPR139 wild type (WT) receptor construct, whereas N271A^7×38^ was 53% expressed compared to WT^16^. There was no measurable Ca^2+^ response from **AC4** stimulation of mutant F109A^3×33^ and N271A^7×38^, and only a weak Ca^2+^ response (17 ± 2% of WT, n = 3) from F109L^3×33^ at the highest tested concentration (100 μM) of **AC4** (Fig. 4). **AC4** had a 12-fold lower EC_50_ on W241H^6×48^ than on WT, with a greatly reduced capacity for receptor activation (E_max_ = 37 ± 3% of WT, n = 3). This suggests that although the structure of **AC4** is unique compared to **1a** and **7c**, it still occupies, at least partly, the same binding site as these known reference ligands.

### Screening and pharmacological characterization of GPR139 compound analogs

**Figure 5:**
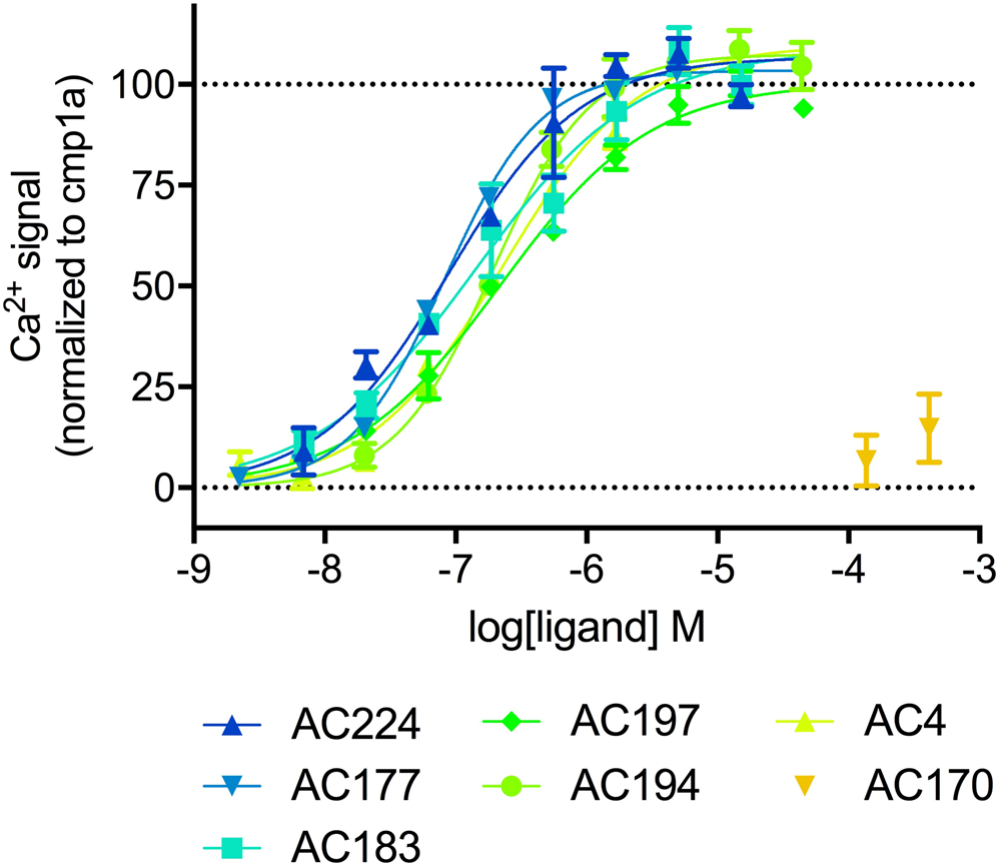
Ca^2+^ mobilization concentration response curves of the most potent **AC4** analogs and the inactive analog **AC170** in CHO-GPR139 cells. The graphs are mean ± S.D. of representative concentration-response curves out of at least three independent experiments performed in duplicate. Data are normalized to the Ca^2+^ response of buffer (0%) and 8 μM **1a** (100%).

To validate these observations and better define the structure-activity relationships of the novel agonists’ pharmacophores, 160 analogs of these initial hits were purchased and tested in Ca^2+^ mobilization assays. From this new analog library we identified 36 novel GPR139 agonists with potencies in the nanomolar range (Fig. 5, Table 1), 36 weak agonists with potencies between 1–10 μM, and a further 11 very weak agonists with EC_50_ values between 10–50 μM, respectively (Supplementary Fig. S2, Supplementary Table S2). Interestingly, a close analog of **AC4**, designated **AC170**, was completely inactive (Fig. 5, Supplementary Table S2).

**Table 1:** Potency and maximal response of the 36 identified GPR139 agonists in calcium mobilization assay in CHO-GPR139 cells.

ID	Enamine ID	EC_50_ μM	pEC_50_ ± S.E.M.	E_max_ ± S.E.M.	n	Matches*
**AC224**	Z30029744	0.09	7.03 ± 0.02	106 ± 1	3	6
**AC177**	Z1618891443	0.09	7.02 ± 0.11	103 ± 1	4	5
**AC183**	Z352587398	0.18	6.74 ± 0.19	110 ± 10	3	5
**AC197**	Z16669083	0.19	6.71 ± 0.14	103 ± 1	3	5
**AC194**	Z18052622	0.21	6.68 ± 0.02	106 ± 4	3	5
**AC4**	Z446416294	0.22	6.66 ± 0.06	116 ± 3	5	3
**AC225**	Z13589494	0.28	6.56 ± 0.05	120 ± 5	3	5
**AC178**	Z1618764723	0.28	6.55 ± 0.07	97 ± 5	3	5
**AC209**	Z18053107	0.29	6.54 ± 0.17	107 ± 3	4	5
**AC82**	Z352587564	0.29	6.54 ± 0.08	102 ± 6	3	4
**AC202**	Z16669106	0.34	6.47 ± 0.09	102 ± 2	3	4
**AC233**	Z14078826	0.34	6.47 ± 0.19	80 ± 6	3	5
**AC191**	Z18052722	0.35	6.46 ± 0.16	99 ± 2	3	5
**AC243**	Z14078663	0.35	6.45 ± 0.11	86 ± 5	3	4
**AC206**	Z16668711	0.38	6.42 ± 0.06	94 ± 3	3	5
**AC234**	Z14075523	0.38	6.42 ± 0.07	99 ± 3	3	5
**AC7**	Z16668610	0.39	6.41 ± 0.12	112 ± 5	4	4
**AC186**	Z14077796	0.41	6.39 ± 0.12	89 ± 5	3	4
**AC241**	Z165543100	0.42	6.38 ± 0.06	88 ± 3	3	5
**AC229**	Z14075450	0.43	6.36 ± 0.16	95 ± 2	4	4
**AC205**	Z18052636	0.44	6.36 ± 0.14	100 ± 3	3	5
**AC200**	Z18052594	0.46	6.34 ± 0.11	111 ± 5	3	5
**AC220**	Z16668660	0.49	6.31 ± 0.01	103 ± 3	3	5
**AC230**	Z14075785	0.49	6.31 ± 0.27	89 ± 1	3	4
**AC137**	Z16669312	0.54	6.27 ± 0.08	92 ± 3	3	5
**AC77**	Z336570670	0.60	6.22 ± 0.17	102 ± 4	3	5
**AC210**	Z18052723	0.63	6.20 ± 0.24	115 ± 8	4	5
**AC123**	Z54783382	0.74	6.13 ± 0.14	104 ± 1	3	5
**AC187**	Z14077801	0.74	6.13 ± 0.22	102 ± 5	4	4
**AC192**	Z16669294	0.75	6.12 ± 0.13	95 ± 1	3	4
**AC14**	Z336589160	0.78	6.11 ± 0.12	93 ± 3	3	5
**AC138**	Z16668376	0.79	6.10 ± 0.14	100 ± 5	4	5
**AC134**	Z16669311	0.80	6.09 ± 0.15	103 ± 5	4	5
**AC208**	Z18053097	0.88	6.05 ± 0.60	134 ± 26	3	5
**AC76**	Z336570672	0.94	6.03 ± 0.10	97 ± 4	3	5
**AC136**	Z16669296	0.99	6.01 ± 0.03	67 ± 6	3	5

The data is based on at least three independent full concentration-response curves in duplicate, normalized to buffer (0%) and 8 μM **1a** (100%). *Number of matching pharmacophore elements from Supplementary Fig. S3.

**Figure 6:**
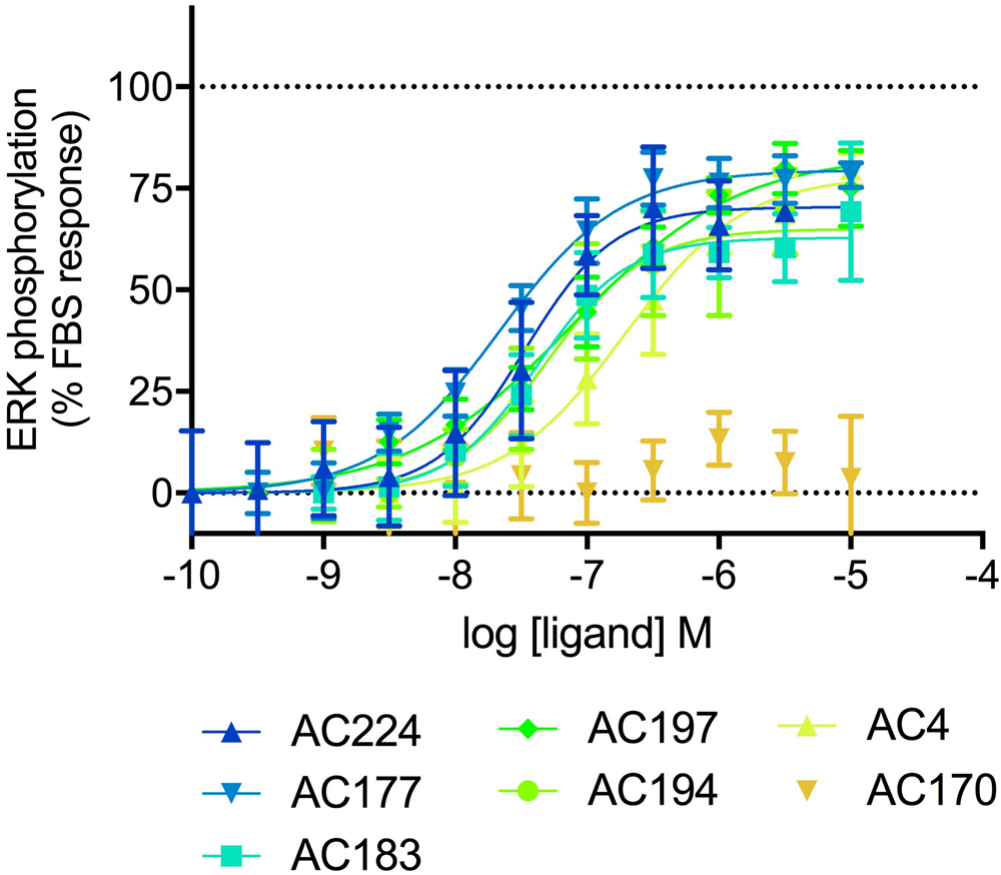
ERK-phosphorylation responses of the most potent compounds identified and the inactive analog **AC170** in CHO-GPR139 cells. Data points represent the mean ± SEM of at least three experiments performed in triplicate. For detailed results including results for reference agonists **1a**, **7c** and L-tryptophan, refer to Table 2.

The lead compound **AC4** and the 5 most potent analogs as well as the inactive analog **AC170** and three reference agonists **1a**, **7c** and L-tryptophan were tested in an orthologonal ERK phosphorylation assay to further assess GPR139 activity. All compounds except **AC170** led to robust, concentration-dependent ERK activation responses with similar potencies (Fig. 6, Table 2) and potency rank-order to the Ca^2+^ mobilization assay results (Figs 1 and 5, Table 1).

**Table 2:** New GPR139 agonists activate ERK with similar rank order of potency.

ID	Enamine ID	EC_50_ μM	pEC_50_ ± SEM	E_max_ ± SEM	n
Compound **7c**	—	0.013	7.89 ± 0.15	89.08 ± 4.38	3
Compound **1a**	—	0.028	7.55 ± 0.14	81.55 ± 4.12	6
L-tryptophan	—	770	3.12 ± 0.26	70.62 ± 8.59	2
**AC224**	Z30029744	0.031	7.51 ± 0.28	69.37 ± 8.01	3
**AC177**	Z1618891443	0.022	7.65 ± 0.13	79.10 ± 3.19	5
**AC183**	Z352587398	0.039	7.41 ± 0.25	64.38 ± 5.28	5
**AC197**	Z16669083	0.94	7.03 ± 0.14	78.70 ± 4.05	5
**AC194**	Z18052622	0.039	7.41 ± 0.30	62.32 ± 6.66	3
**AC** **4**	Z446416294	0.18	6.75 ± 0.21	77.22 ± 6.53	3
**AC** **170**	Z838953070	n.d.		no activity	3

Agonist potency (EC_50_ and pEC_50_) and efficacy (E_max,_ expressed relative to FBS positive control) of the 6 most potent new GPR139 agonists and structurally similar inactive analogue **AC170**. Previously reported GPR139 agonists (compound **1a**, compound **7c** and L-tryptophan) are reported for comparison. n.d. (not determined, as concentration-response curves could not be generated).

### AC4 presents a novel GPCR139 agonist scaffold

Our 36 novel GPR139 agonists were compared to the salient structural elements in our previously published GPR139 pharmacophore model^13^. Only the most potent analog compound, **AC224**, matched all 6 of our designated pharmacophore elements (Table 1, Supplementary Fig. S3). In contrast to the majority of new agonists that matched at least four of the six pharmacophore elements, **AC4** featured an alternative structure, which only matched three of the elements (Supplementary Fig. S3). Thus, **AC4** presents a novel chemical scaffold containing only one terminal aromatic ring, a carboxylic acid at the ligand’s opposite terminal, and an oxadiazole moiety in the linker region (Fig. 1).

## Discussion

We performed a screening campaign using a diverse chemical library and identified 36 potent GPR139 agonists, among them **AC4** which introduces a unique scaffold not shared by any previously described GPR139 agonists. **AC4** displays an EC_50_ value of 220 nM and a similar maximal response as the previously published agonist **1a**^11^. Similar to the aromatic amino acid agonists (L-tryptophan and L-phenylalanine)^5,7^, the chemical structure of **AC4** presents a terminal aromatic ring. This is in contrast to **1a** and **7c** that feature aromatic systems on each end. Additionally, **AC4** contains a carboxylate moiety on its terminal ring system (Fig. 1). Despite the lack of aromaticity at both ends of the molecule, the aliphatic ring in **AC4** fits well into the suggested hydrophobic pharmacophore element (Supplementary Fig. S3), previously seen in all reported analogs^13^.

Another unprecedented feature of **AC4** is its lack of a hydrogen bond donor in its linker region. Instead, **AC4** displays a unique cyclic aromatic 1,3,4-oxadiazole ring. In general, our presented analogues (Fig. S1) demonstrate a new linker cyclization exemplified by an imidazolidine-2,4-dione ring, which can substitute for the polar linkers seen in other GPR139 ligand examples^13^. We previously reported that cyclization in the linker 2 position, as shown in the Hitchcock *et al*. compound series, is beneficial, whereas cyclization at the linker 5 and 6 positions is unfavorable^13^. Here, we report 23 nanomolar GPR139 agonists that represent the first compounds with a novel linker cyclization between atoms 3 and 5 (**AC123**, **AC134**, **AC136, AC137**, **AC138**, **AC186**, **AC187**, **AC191**, **AC192**, **AC197**, **AC200**, **AC202**, **AC205**, **AC206**, **AC208**, **AC209**, **AC210**, **AC224**, **AC225**, **AC229**, **AC233**, **AC234**, and **AC241**), as well as a novel aromatic cyclic linker (**AC4**) (Supplementary Fig. S3, Supplementary Table S2).

It was interesting to note that a commercially available analog of **AC4**, designated **AC170**, did not evoke a GPR139 response in either Ca^2+^ mobilization or ERK phosphorylation assays (Figs 1, 5 and 6, Table 2 and Supplementary Table S2). This could be due to the change in the chemical nature of the substituent on the aromatic ring from being weak electron-withdrawing (halogen on **AC4**) into a strong electron-withdrawing group (nitrile on **AC170**). Our previous SAR analysis suggests that the size of both groups can be tolerated in other described ligands^13^. Moreover, due to the difference in electronegativity, the ether oxygen in **AC170** could be playing another role in weakening the aromatic ring character more than the thioether sulfur in **AC4**. Taken together, these features could explain the observed loss of activity.

Based on the available data, we suggested in our previous SAR analysis that a second terminal aromatic group contributes to the high potency of the most potent analogues **1a**, **7c**, and **39** from Shi *et al*., Dvorak *et al*., and Hitchcock *et al*., respectively^13^. Interestingly, our ligands **AC4**, **AC7**, **AC76**, **AC77**, **AC82, AC177**, **AC178**, **AC183**, **AC186**, **AC187**, **AC192**, **AC194**, **AC220**, **AC229**, and **AC230** all lack a second terminal aromatic ring (Supplementary Fig. S3), yet they all possess EC_50_ values in the nanomolar range (90–990 nM). Thus, this series of new agonists demonstrate that one aromatic ring can be sufficient for potent GPR139 activity.

Our study also provided the first detailed pharmacological analyses of GPR139-mediated ERK phosphorylation using a state-of-the-art TR-FRET based assay, and show that the receptor is strongly coupled to that pathway (Fig. 6, Table 2). This correlates with and greatly expands a previous report showing that a high concentration of L-tryptophan and L-phenylalanine increased ERK phosphorylation in a Western blot assay^5^. Recent studies have shown that biased agonists can differentially activate intracellular calcium and ERK phosphorylation responses, which potentially can lead to different physiological responses and therapeutic effects^17^. It was thus of interest to determine if the most potent agonists identified in the present study as well as reference agonists **1a**, **7c** and L-tryptophan displayed such biased signaling or activate both pathways equally well. Our data showed that all compounds shows similar potencies and potency rank-order in both pathways, and thus are not biased with respect to these signaling pathways/endpoints (Figs 1, 5 and 6, Tables 1 and 2).

Our mutagenesis data, suggest that F109^3×33^, W241^6×48^ and N271^7×38^ are important for GPR139 activation by **AC4**, as previously shown for **1a** and **7c**^16^. This provides strong evidence that the novel scaffold of **AC4** is equipotent, and it also shares the same binding site as the other GPR139 agonists, despite the differences between their chemical structures.

Given our successful identification of a new agonist scaffold, it was striking that we did not identify any GPR139 antagonists in our screen. It appears that these have been challenging given the discrepancy between published findings on small molecule agonists versus antagonists that target GPR139^10,12–16^. The development of more potent and selective antagonists remains an area of great interest, and would be of particular value for *in vivo* studies to delineate the biological function of the receptor.

In summary, we have identified 36 novel GPR139 agonists with nanomolar potency. Interestingly, we discovered **AC4**, a compound with novel structural features that distinguish it from other known GPR139 agonists. We showed via mutagenesis that **AC4** activates GPR139 in a similar manner as previously published agonists despite its different chemical structure. We also identified 5 more potent analogs of the original hits that share a similar rank order of potency in both Ca^2+^ mobilization and ERK assays. All together this data adds to our knowledge about surrogate agonists for GPR139.

## Methods

### Diverse compound library generation

Compound **1a** was kindly provided by H. Lundbeck A/S, Denmark, and carbamoylcholine chloride (carbachol) was obtained from Sigma-Aldrich (C4382). The screening compound library (Enamine, Kiev, Ukraine) consisted of 4,000 compounds. The compounds were selected from a pool of 30,300 compounds from the Enamine screening compound catalogue based on the four following filters; (1) Molecular weight (g/mol) 200–500, (2) Hydrogen bond donors 1–5, (3) Hydrogen bond acceptors 2–10, (4) Maximum calculated logP = 4.5. These criteria yielded 7,339 unique structures. Similar compounds were removed based on their Tanimoto similarity score^18^, in order to obtain a structurally diverse library with 4,000 compounds.

### Cells and cell culture

All compounds were tested on a CHO-k1 cell line stably expressing GPR139 (CHO-GPR139)^11^ kindly provided by H. Lundbeck A/S, Denmark. GPR139-specific responses were assessed using a CHO-k1 cell line stably expressing the muscarinic acetylcholine receptor M1 (CHO-M1) (The Missouri S&T cDNA Resource Center, # CEM100TN00). The CHO-GPR139 cells were grown in Dulbecco’s modified Eagle’s medium (DMEM) F12-Kaighn’s (ThermoFisher Scientific # 21127) supplemented with 10% dialyzed fetal bovine serum (United States origin; ThermoFisher Scientific # 26400), 1% GlutaMAX-I (100X) (ThermoFisher Scientific # 35050061), and 100 units/mL penicillin and 100 µg/mL streptomycin (ThermoFisher Scientific # 15140122) and 1 mg/mL geneticin (ThermoFisher Scientific # 11811031). The CHO-M1 cells were grown in Ham’s F12 (ThermoFisher Scientific # 21765) supplemented with 10% fetal bovine serum (South American origin; ThermoFisher Scientific # 10270) + 100 units/mL penicillin and streptomycin and 0.25 mg/mL geneticin.

### Primary screening using calcium mobilization

The Fluo-4 Ca^2+^-assay was performed as described previously^13^, with minor modifications. After 1 hour incubation with the Fluo-4 dye, the CHO-GPR139 cells were washed with 100 μL HEPES buffer (Hank’s Balanced Salt Solution (HBBS, ThermoFisher Scientific # 14175053) supplemented with 20 mM HEPES, 1 mM MgCl2, 1 mM CaCl_2_, pH = 7.4) and the compound library was then added in concentrations between 25–63 μM dissolved in HEPES buffer (supplemented with 2.5 mM probenecid and 1% DMSO) and pre-incubated for 10 min at 37 °C. 33 μL of 3.2 μM **1a** were added automatically after baseline measurements. Intracellular Ca^2+^ changes were recorded on a NOVOstar instrument (BMG Labtech) at 37 °C with an excitation filter of 485 nm and an emission filters 520 nm. This set-up allowed us to identify both antagonists and agonists (Fig. 2).

### Hit validation and analog purchases

The primary screening of 4,000 diverse screening compounds on GPR139, led us to identify 108 initial actives in GPR139-expressing cells. These 108 compounds inhibited the Ca^2+^ response induced by 800 nM **1a** (≈EC_80_) by more than 75%. We excluded compounds that had (1) an effect on another simultaneously tested GPCR (data not shown), (2) reactive groups, or (3) poor predicted solubility. 52 of the active compounds were re-purchased for validation, which were tested at three concentrations (100, 50 and 25 μM) using the same methodology as in the screening campaign. Out of the 52 compounds, 13 compounds had no effect (excluded as false positives), 28 compounds elicited an equal response in GPR139 and muscarinic acetylcholine receptor M1 cells and were designated as not selective, and 11 compounds were identified as viable candidates (**AC4**, **AC7**, **AC8**, **AC9**, **AC10**, **AC11**, **AC12**, **AC13**, **AC14**, **AC16**, and **AC37**) (Supplementary Fig. S1). None of the 11 hits promoted calcium mobilization in the M1 receptor cells (data not shown). The 11 hits were then screened in pure agonist mode (i.e., without the presence of **1a**) to evaluate if they were agonists or antagonists (Fig. 3 and Supplementary Table S1). All 11 hits were agonists.

### Calcium mobilization assay for concentration-response curves of hits and analogs

#### Agonist mode

Each compound, for which a concentration response curve was made, was tested at least three times in duplicate measurements. After the incubation with the Fluo-4 dye and a wash, 100 μL HEPES buffer (supplemented with 2.5 mM probenecid) was added to each well and incubated for 10 minutes at 37 °C before recording. 33 μL of compounds (4× concentrated) were added automatically after baseline measurements. All concentration response curves of the 11 hits and 160 analogs were measured on a FlexStation 3 Benchtop Multi-Mode Microplate Reader (Molecular Devices) at 37 °C with an excitation filter of 485 nm and emission at 525 nm.

#### Antagonist mode

When screening the compounds as antagonists they were pre-incubated for 10 min as in the HTS. 33 μL of 3.2 μM **1a** on the CHO-GPR139 cells or 20 μM carbachol on the CHO-M1 cells were added automatically after baseline measurements. These data were measured on the FlexStation.

### Concentration-response curves of analogs in phospho-ERK assay

Phospho-ERK measurements were made using the Advanced Phospho-ERK1/2 (Thr202/Tyr204) HTRF kit by Cisbio (Codolet, France). Approximately 24 hours prior to the experiment, CHO-GPR139 cells were plated at 50,000 cells/well onto a 96-well plate and cultured as detailed above. Approximately 6 hours later, the media was aspirated, replaced with 50 μL serum-free media, and the cells were allowed to serum starve overnight. The day of the experiment, serial dilutions of the test compounds and controls were made in serum-free media at 2x final concentration. 50 μL of test compounds were added directly to the appropriate wells for 5 minute stimulation at 37 °C. At this point, the media and compounds were rapidly aspirated, lysis/blocking buffer was added, and cells were lysed on a shaker for 30 minutes at room temperature. Lysates were combined with detection solution, incubated for 4 hours, and read on an Envision plate reader measuring emission at 665 and 620 nm.

### Mutational studies

The mutational studies were performed as previously described^16^.

### Data analysis

The Ca^2+^ and ERK responses were normalized as indicated and the concentration response curves were fitted by Prism 6 software (GraphPad) using nonlinear regression in a sigmoidal model with variable slope, as has been previously described^8^.

### Ligand preparation and pharmacophore matching

LigPrep was used for ligand preparation^19^. Macromodel was employed to do the conformational analysis on ligands using default settings^20^. This includes using the OPLS_3 force filed^21^, and the Monte Carlo approach for sampling the different conformations. The global energy non-collapsed conformation of the ligands was picked for further analysis and superposition. Phase was used to match the pharmacophore to the herein presented analogues with default settings^22–24^.

## Supplementary information

Supplementary Information

## Data Availability

The authors declare that all data supporting the findings of this study are available within the article and Supplementary Information, or are available from corresponding authors upon request.
