# Supplementary material for: Identification of a novel scaffold for a small molecule GPR139 receptor agonist
Source: Sci Rep. 2019 Mar 7;9:3802. doi: 10.1038/s41598-019-40085-9 (PMC6405842; doi:10.1038/s41598-019-40085-9)
Supplement: Supplementary file 1 — Supplementary Information [file 41598_2019_40085_MOESM1_ESM.docx]

**Supplementary information**

**Identification of a novel scaffold for a small molecule GPR139 receptor agonist**

Anne Cathrine Nøhr^1†^, Mohamed A. Shehata^1†^, Daniel Palmer^2^, Rina Pokhrel^1^, Maria Vallianou^1^, Simon R. Foster^1^, Patrick R. Gentry^1^, David E. Gloriam^1§^*, and Hans Bräuner-Osborne^1§^*.

^1^Department of Drug Design and Pharmacology, Faculty of Health and Medical Sciences, University of Copenhagen, Universitetsparken 2, 2100 Copenhagen, Denmark.

^2^Center for Evolutionary Chemical Biology, Department of Chemistry, Faculty of Science, University of Copenhagen, Universitetsparken 5, 2100 Copenhagen, Denmark

^†^,^§^ These authors contributed equally

* Corresponding authors. E-mail address: [david.gloriam@sund.ku.dk](mailto:david.gloriam@sund.ku.dk) (D.E.G. computational chemistry), [hbo@sund.ku.dk](mailto:hbo@sund.ku.dk) (H.B.-O. pharmacology)

**Supplementary Figure S1.** Chemical structures of the 11 GPR139 hits identified in the primary screen of a diverse 4000 compound library. The compound names and EC_50_ is (in μM) for calcium mobilization are given below the structure (n.d., not determined).

**Supplementary Figure S2.** Chemical structures for GPR139 ligands identified in this study, in order of potency in calcium mobilization. The compound names are given below the structure along with the EC_50_ (in μM).


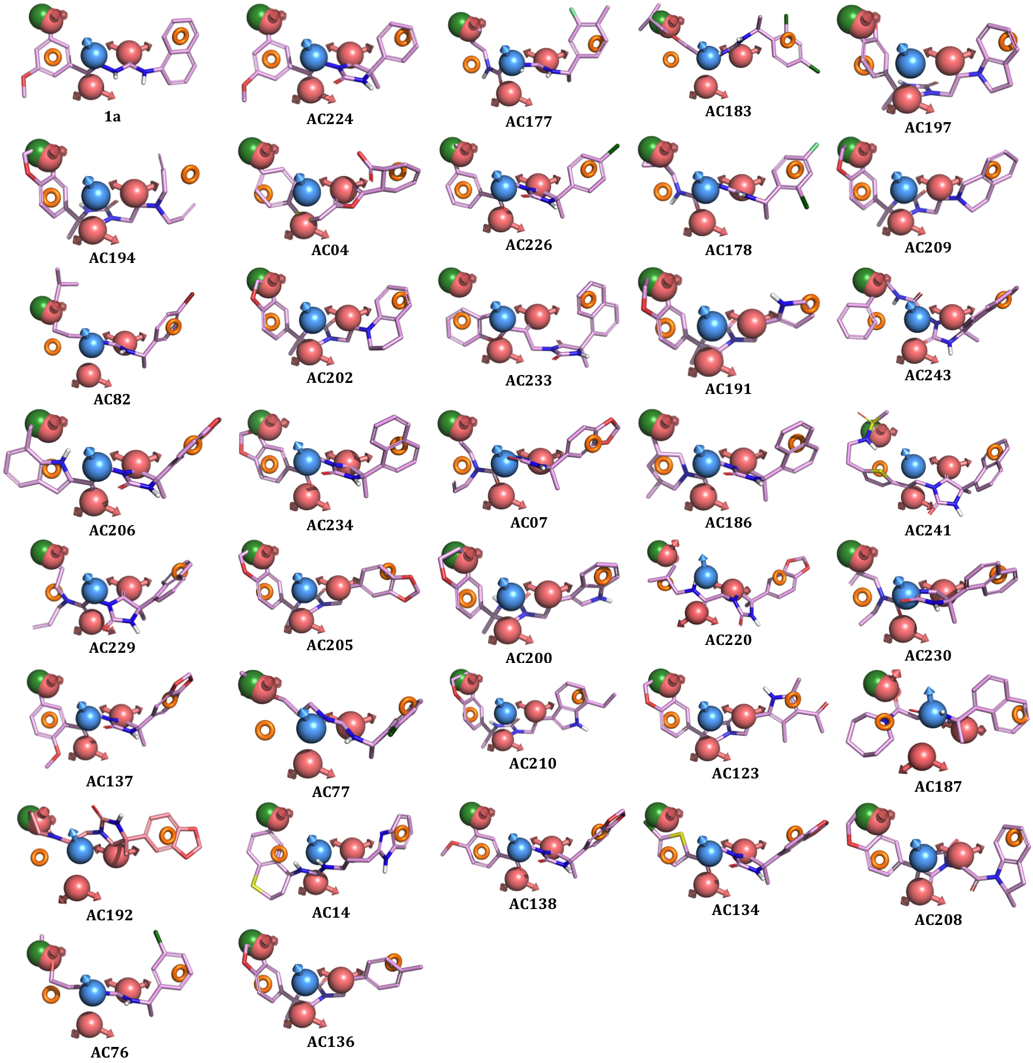


**Supplementary Figure S3.** Matching of 36 identified GPR139 agonists to a common pharmacophore model for the previously published agonists^1^. Ligands are shown pink, and for clarity, labels and exclusion volumes are not displayed. The ligands show an overall good fit to the different features in the pharmacophore. The reference compound **1a** is shown on the top left panel for comparison.

**Supplementary Table S1. GPR139 agonists from diverse chemical library of 4000 compounds.** Agonist potency (EC_50_ and pEC_50_) and efficacy (E_max_) of the 11 hits in the primary screen on GPR139 normalized to compound **1a**. n.d. (not determined, as complete concentration-response curves could not be generated).

| **Compound ID** | **Enamine ID** | **EC_50_ μM** | **pEC_50_ ± S.E.M.** | **E_max_ ± S.E.M.** | **n** |
| --- | --- | --- | --- | --- | --- |
| AC4 | Z446416294 | 0.22 | -6.66 ± 0.06 | 115 ± 3 | 5 |
| AC7 | Z16668610 | 0.39 | -6.41 ± 0.12 | 112 ± 5 | 4 |
| AC14 | Z336589160 | 0.78 | -6.11 ± 0.12 | 93 ± 3 | 3 |
| AC37 | Z353305998 | 2.80 | -5.55 ± 0.19 | 105 ± 8 | 3 |
| AC10 | Z25729925 | 2.83 | -5.55 ± 0.03 | 89 ± 5 | 2 |
| AC12 | Z336572328 | 3.74 | -5.43 ± 0.19 | 116 ± 8 | 3 |
| AC11 | Z52080241 | 7.07 | -5.15 ± 0.12 | 33 ± 3 | 3 |
| AC16 | Z89591025 | 11.89 | -4.92 ± 0.12 | 44 ± 4 | 5 |
| AC13 | Z212164676 | 20.72 | -4.68 ± 0.13 | 90 ± 5 | 5 |
| AC8 | Z16668350 | n.d. | n.d. | 30 ± 6 at 39 μM | 4 |
| AC9 | Z65671638 | n.d. | n.d. | 62 ± 5 at 39 μM | 3 |

**Supplementary Table S2. New GPR139 agonists and analogues.** Agonist potency (EC_50_ and pEC_50_) and efficacy (E_max_) of the 11 primary hits and 160 purchased analogues in calcium mobilization assay. The compounds are sorted according to their potency on GPR139.

| **ID** | **Enamine no** | **EC_50_ μM** | **pEC_50_ ± SEM** | | | **E_max_ ± SEM** | | | | | | **n** | **Note** |
| --- | --- | --- | --- | --- | --- | --- | --- | --- | --- | --- | --- | --- | --- |
| AC224 | Z30029744 | 0.09 | -7.03 | ± | 0.02 | 106 | ± | 1 |  |  |  | 3 |  |
| AC177 | Z1618891443 | 0.09 | -7.02 | ± | 0.11 | 103 | ± | 1 |  |  |  | 4 |  |
| AC183 | Z352587398 | 0.18 | -6.74 | ± | 0.19 | 110 | ± | 10 |  |  |  | 3 |  |
| AC197 | Z16669083 | 0.19 | -6.71 | ± | 0.14 | 103 | ± | 1 |  |  |  | 3 |  |
| AC194 | Z18052622 | 0.21 | -6.68 | ± | 0.02 | 106 | ± | 4 |  |  |  | 3 |  |
| AC004 | Z446416294 | 0.22 | -6.66 | ± | 0.06 | 116 | ± | 3 |  |  |  | 5 |  |
| AC225 | Z13589494 | 0.28 | -6.56 | ± | 0.05 | 120 | ± | 5 |  |  |  | 3 | Nonspecific ≥ 10 μM |
| AC178 | Z1618764723 | 0.28 | -6.55 | ± | 0.07 | 97 | ± | 5 |  |  |  | 3 |  |
| AC209 | Z18053107 | 0.29 | -6.54 | ± | 0.17 | 107 | ± | 3 |  |  |  | 4 |  |
| AC082 | Z352587564 | 0.29 | -6.54 | ± | 0.08 | 102 | ± | 6 |  |  |  | 3 |  |
| AC202 | Z16669106 | 0.34 | -6.47 | ± | 0.09 | 102 | ± | 2 |  |  |  | 3 |  |
| AC233 | Z14078826 | 0.34 | -6.47 | ± | 0.19 | 80 | ± | 6 |  |  |  | 3 |  |
| AC191 | Z18052722 | 0.35 | -6.46 | ± | 0.16 | 99 | ± | 2 |  |  |  | 3 |  |
| AC243 | Z14078663 | 0.35 | -6.45 | ± | 0.11 | 86 | ± | 5 |  |  |  | 3 |  |
| AC206 | Z16668711 | 0.38 | -6.42 | ± | 0.06 | 94 | ± | 3 |  |  |  | 3 | Nonspecific ≥ 100 μM |
| AC234 | Z14075523 | 0.38 | -6.42 | ± | 0.07 | 99 | ± | 3 |  |  |  | 3 |  |
| AC007 | Z16668610 | 0.39 | -6.41 | ± | 0.12 | 112 | ± | 5 |  |  |  | 4 |  |
| AC186 | Z14077796 | 0.41 | -6.39 | ± | 0.12 | 89 | ± | 5 |  |  |  | 3 |  |
| AC241 | Z165543100 | 0.42 | -6.38 | ± | 0.06 | 88 | ± | 3 |  |  |  | 3 |  |
| AC229 | Z14075450 | 0.43 | -6.36 | ± | 0.16 | 95 | ± | 2 |  |  |  | 4 |  |
| AC205 | Z18052636 | 0.44 | -6.36 | ± | 0.14 | 100 | ± | 3 |  |  |  | 3 |  |
| AC200 | Z18052594 | 0.46 | -6.34 | ± | 0.11 | 111 | ± | 5 |  |  |  | 3 |  |
| AC220 | Z16668660 | 0.49 | -6.31 | ± | 0.01 | 103 | ± | 3 |  |  |  | 3 |  |
| AC230 | Z14075785 | 0.49 | -6.31 | ± | 0.27 | 89 | ± | 1 |  |  |  | 3 |  |
| AC137 | Z16669312 | 0.54 | -6.27 | ± | 0.08 | 92 | ± | 3 |  |  |  | 3 |  |
| AC077 | Z336570670 | 0.60 | -6.22 | ± | 0.17 | 102 | ± | 4 |  |  |  | 3 |  |
| AC210 | Z18052723 | 0.63 | -6.20 | ± | 0.24 | 115 | ± | 8 |  |  |  | 4 |  |
| AC123 | Z54783382 | 0.74 | -6.13 | ± | 0.14 | 104 | ± | 1 |  |  |  | 3 | Nonspecific ≥ 10 μM |
| AC187 | Z14077801 | 0.74 | -6.13 | ± | 0.22 | 102 | ± | 5 |  |  |  | 4 |  |
| AC192 | Z16669294 | 0.75 | -6.12 | ± | 0.13 | 95 | ± | 1 |  |  |  | 3 |  |
| AC014 | Z336589160 | 0.78 | -6.11 | ± | 0.12 | 93 | ± | 3 |  |  |  | 3 |  |
| AC138 | Z16668376 | 0.79 | -6.10 | ± | 0.14 | 100 | ± | 5 |  |  |  | 4 |  |
| AC134 | Z16669311 | 0.80 | -6.09 | ± | 0.15 | 103 | ± | 5 |  |  |  | 4 | Nonspecific ≥ 40 μM |
| AC208 | Z18053097 | 0.88 | -6.05 | ± | 0.60 | 134 | ± | 26 |  |  |  | 3 | Nonspecific ≥ 30 μM |
| AC076 | Z336570672 | 0.94 | -6.03 | ± | 0.10 | 97 | ± | 4 |  |  |  | 3 |  |
| AC136 | Z16669296 | 0.99 | -6.01 | ± | 0.03 | 67 | ± | 6 |  |  |  | 3 |  |
| AC196 | Z16668420 | 1.00 | -6.00 | ± | 0.09 | 65 | ± | 9 |  |  |  | 3 |  |
| AC228 | Z14076022 | 1.06 | -5.98 | ± | 0.11 | 63 | ± | 5 |  |  |  | 3 |  |
| AC222 | Z16668613 | 1.16 | -5.94 | ± | 0.16 | 98 | ± | 3 |  |  |  | 4 |  |
| AC198 | Z18052530 | 1.19 | -5.93 | ± | 0.29 | 102 | ± | 23 |  |  |  | 4 | Nonspecific ≥ 30 μM |
| AC193 | Z18053314 | 1.22 | -5.92 | ± | 0.04 | 99 | ± | 1 |  |  |  | 3 | Nonspecific ≥ 30 μM |
| AC189 | Z16669284 | 1.30 | -5.89 | ± | 0.05 | 100 | ± | 3 |  |  |  | 3 |  |
| AC078 | Z336570782 | 1.31 | -5.88 | ± | 0.03 | 101 | ± | 3 |  |  |  | 3 |  |
| AC061 | Z362607888 | 1.32 | -5.88 | ± | 0.11 | 109 | ± | 5 |  |  |  | 3 |  |
| AC059 | Z956169746 | 1.44 | -5.84 | ± | 0.03 | 105 | ± | 5 |  |  |  | 3 |  |
| AC133 | Z16668360 | 1.46 | -5.84 | ± | 0.05 | 103 | ± | 4 |  |  |  | 3 |  |
| AC159 | Z81973167 | 1.63 | -5.79 | ± | 0.13 | 114 | ± | 10 |  |  |  | 3 | Nonspecific ≥ 10 μM |
| AC201 | Z16668616 | 1.80 | -5.75 | ± | 0.04 | 96 | ± | 5 |  |  |  | 3 |  |
| AC155 | Z51069605 | 1.85 | -5.73 | ± | 0.04 | 97 | ± | 5 |  |  |  | 4 |  |
| AC238 | Z14074724 | 1.85 | -5.73 | ± | 0.00 | 94 | ± | 1 |  |  |  | 3 |  |
| AC236 | Z14078242 | 1.87 | -5.73 | ± | 0.14 | 56 | ± | 7 |  |  |  | 3 |  |
| AC221 | Z97822815 | 1.88 | -5.73 | ± | 0.16 | 103 | ± | 4 |  |  |  | 3 |  |
| AC090 | Z26239702 | 1.90 | -5.72 | ± | 0.10 | 106 | ± | 2 |  |  |  | 3 |  |
| AC185 | Z14073685 | 1.91 | -5.72 | ± | 0.15 | 84 | ± | 1 |  |  |  | 3 |  |
| AC058 | Z1518175860 | 1.99 | -5.70 | ± | 0.16 | 97 | ± | 4 |  |  |  | 3 |  |
| AC144 | Z13598211 | 2.06 | -5.69 | ± | 0.05 | 94 | ± | 3 |  |  |  | 3 |  |
| AC158 | Z16668624 | 2.08 | -5.68 | ± | 0.14 | 114 | ± | 4 |  |  |  | 3 | Nonspecific ≥ 30 μM |
| AC087 | Z25711636 | 2.08 | -5.68 | ± | 0.07 | 102 | ± | 1 |  |  |  | 3 |  |
| AC135 | Z16669300 | 2.14 | -5.67 | ± | 0.06 | 94 | ± | 3 |  |  |  | 3 | Nonspecific ≥ 10 μM |
| AC037 | Z353305998 | 2.80 | -5.55 | ± | 0.19 | 105 | ± | 8 |  |  |  | 3 |  |
| AC231 | Z14075770 | 2.81 | -5.55 | ± | 0.03 | 77 | ± | 1 |  |  |  | 3 |  |
| AC010 | Z25729925 | 2.83 | -5.55 | ± | 0.03 | 89 | ± | 5 |  |  |  | 2 | Poor solubility in polar solvents |
| AC245 | Z220020234 | 3.09 | -5.51 | ± | 0.07 | 66 | ± | 4 |  |  |  | 4 |  |
| AC141 | Z134497312 | 3.18 | -5.50 | ± | 0.05 | 97 | ± | 2 |  |  |  | 3 |  |
| AC190 | Z18053003 | 3.56 | -5.45 | ± | 0.12 | 107 | ± | 3 |  |  |  | 3 |  |
| AC012 | Z336572328 | 3.74 | -5.43 | ± | 0.19 | 116 | ± | 8 |  |  |  | 3 |  |
| AC216 | Z51069607 | 4.23 | -5.37 | ± | 0.13 | 97 | ± | 4 |  |  |  | 3 |  |
| AC161 | Z13602182 | 6.40 | -5.19 | ± | 0.00 | 82 | ± | 4 |  |  |  | 3 |  |
| AC195 | Z16668468 | 6.53 | -5.19 | ± | 0.12 | 101 | ± | 2 |  |  |  | 3 |  |
| AC011 | Z52080241 | 7.07 | -5.15 | ± | 0.12 | 33 | ± | 3 |  |  |  | 3 |  |
| AC080 | Z352585756 | 8.07 | -5.09 | ± | 0.08 | 92 | ± | 6 |  |  |  | 4 |  |
| AC168 | Z98685706 | 9.77 | -5.01 | ± | 0.21 | 77 | ± | 3 |  |  |  | 3 |  |
| AC211 | Z51069604 | 10.25 | -4.99 | ± | 0.10 | 99 | ± | 4 |  |  |  | 3 |  |
| AC053 | Z1627056500 | 11.35 | -4.95 | ± | 0.04 | 98 | ± | 4 |  |  |  | 3 |  |
| AC016 | Z89591025 | 11.89 | -4.92 | ± | 0.12 | 44 | ± | 4 |  |  |  | 5 |  |
| AC086 | Z25742116 | 12.09 | -4.92 | ± | 0.13 | 101 | ± | 5 |  |  |  | 4 |  |
| AC179 | Z224745274 | 14.10 | -4.85 | ± | 0.11 | 87 | ± | 4 |  |  |  | 3 |  |
| AC091 | Z26239699 | 14.44 | -4.84 | ± | 0.09 | 103 | ± | 6 |  |  |  | 3 |  |
| AC070 | Z434246796 | 14.84 | -4.83 | ± | 0.07 | 89 | ± | 3 |  |  |  | 3 |  |
| AC181 | Z212165778 | 16.08 | -4.79 | ± | 0.14 | 75 | ± | 9 |  |  |  | 3 |  |
| AC013 | Z212164676 | 20.72 | -4.68 | ± | 0.13 | 90 | ± | 5 |  |  |  | 5 |  |
| AC132 | Z50992541 | 21.74 | -4.66 | ± | 0.22 | 113 | ± | 9 |  |  |  | 4 |  |
| AC092 | Z25741355 | 24.03 | -4.62 | ± | 0.18 | 95 | ± | 3 |  |  |  | 4 |  |
| AC071 | Z434243670 | 51.40 | -4.29 | ± | 0.05 | 99 | ± | 5 |  |  |  | 3 |  |
| AC127 | Z13591125 |  |  |  |  | 93 | ± | 5 | at | 398 | μM | 3 | Nonspecific ≥ 400 μM |
| AC128 | Z50992523 |  |  |  |  | 81 | ± | 9 | at | 42 | μM | 3 | Nonspecific ≥ 40 μM |
| AC246 | Z14075393 |  |  |  |  | 79 | ± | 6 | at | 39 | μM | 3 |  |
| AC129 | Z16668522 |  |  |  |  | 77 | ± | 5 | at | 38 | μM | 3 |  |
| AC239 | Z50920447 |  |  |  |  | 77 | ± | 7 | at | 38 | μM | 3 |  |
| AC162 | Z13600425 |  |  |  |  | 77 | ± | 4 | at | 398 | μM | 4 |  |
| AC088 | Z25836605 |  |  |  |  | 74 | ± | 4 | at | 418 | μM | 3 |  |
| AC180 | Z336571232 |  |  |  |  | 74 | ± | 4 | at | 409 | μM | 3 |  |
| AC184 | Z973182218 |  |  |  |  | 74 | ± | 5 | at | 409 | μM | 3 |  |
| AC223 | Z14049733 |  |  |  |  | 73 | ± | 6 | at | 44 | μM | 3 | Nonspecific ≥ 50 μM |
| AC204 | Z16669224 |  |  |  |  | 72 | ± | 5 | at | 15 | μM | 5 | Nonspecific ≥ 16 μM |
| AC244 | Z103028608 |  |  |  |  | 72 | ± | 7 | at | 43 | μM | 3 |  |
| AC219 | Z74348180 |  |  |  |  | 70 | ± | 3 | at | 43 | μM | 4 |  |
| AC232 | Z14075300 |  |  |  |  | 70 | ± | 7 | at | 47 | μM | 4 | Nonspecific ≥ 50 μM |
| AC167 | Z65671627 |  |  |  |  | 70 | ± | 7 | at | 389 | μM | 3 |  |
| AC203 | Z16668558 |  |  |  |  | 70 | ± | 5 | at | 39 | μM | 3 |  |
| AC214 | Z74370273 |  |  |  |  | 69 | ± | 8 | at | 39 | μM | 3 |  |
| AC235 | Z152915884 |  |  |  |  | 68 | ± | 5 | at | 39 | μM | 3 |  |
| AC173 | Z1004823114 |  |  |  |  | 68 | ± | 3 | at | 427 | μM | 3 |  |
| AC217 | Z18052640 |  |  |  |  | 67 | ± | 3 | at | 44 | μM | 3 |  |
| AC062 | Z211829288 |  |  |  |  | 67 | ± | 7 | at | 377 | μM | 3 |  |
| AC079 | Z336572006 |  |  |  |  | 65 | ± | 7 | at | 412 | μM | 4 |  |
| AC140 | Z98892815 |  |  |  |  | 65 | ± | 3 | at | 42 | μM | 3 |  |
| AC182 | Z336644098 |  |  |  |  | 63 | ± | 4 | at | 404 | μM | 3 |  |
| AC237 | Z14076594 |  |  |  |  | 62 | ± | 11 | at | 38 | μM | 3 |  |
| AC009 | Z65671638 |  |  |  |  | 62 | ± | 5 | at | 39 | μM | 3 |  |
| AC143 | Z103285062 |  |  |  |  | 60 | ± | 3 | at | 385 | μM | 3 |  |
| AC213 | Z16668628 |  |  |  |  | 58 | ± | 9 | at | 42 | μM | 3 |  |
| AC142 | Z16668643 |  |  |  |  | 58 | ± | 8 | at | 38 | μM | 3 |  |
| AC073 | Z212162656 |  |  |  |  | 56 | ± | 11 | at | 41 | μM | 4 |  |
| AC150 | Z65773823 |  |  |  |  | 56 | ± | 8 | at | 39 | μM | 5 | Nonspecific ≥ 40 μM |
| AC151 | Z65993362 |  |  |  |  | 55 | ± | 5 | at | 39 | μM | 3 |  |
| AC122 | Z13588583 |  |  |  |  | 54 | ± | 13 | at | 40 | μM | 5 | Nonspecific ≥ 40 μM |
| AC242 | Z73938625 |  |  |  |  | 54 | ± | 9 | at | 41 | μM | 3 |  |
| AC218 | Z18052644 |  |  |  |  | 53 | ± | 3 | at | 43 | μM | 3 |  |
| AC227 | Z14053710 |  |  |  |  | 53 | ± | 6 | at | 44 | μM | 3 | Nonspecific ≥ 50 μM |
| AC146 | Z54068832 |  |  |  |  | 52 | ± | 5 | at | 404 | μM | 3 |  |
| AC240 | Z96789667 |  |  |  |  | 52 | ± | 4 | at | 39 | μM | 3 |  |
| AC072 | Z434243676 |  |  |  |  | 49 | ± | 6 | at | 382 | μM | 3 |  |
| AC068 | Z212164666 |  |  |  |  | 49 | ± | 7 | at | 42 | μM | 3 |  |
| AC063 | Z1130352345 |  |  |  |  | 48 | ± | 4 | at | 387 | μM | 2 |  |
| AC093 | Z1729582545 |  |  |  |  | 47 | ± | 6 | at | 420 | μM | 3 |  |
| AC169 | Z13596662 | - |  |  |  | 46 | ± | 12 | at | 426 | μM | 3 |  |
| AC089 | Z91713589 |  |  |  |  | 45 | ± | 5 | at | 409 | μM | 2 |  |
| AC165 | Z50916152 |  |  |  |  | 44 | ± | 8 | at | 39 | μM | 3 |  |
| AC148 | Z65845221 |  |  |  |  | 44 | ± | 4 | at | 413 | μM | 3 |  |
| AC075 | Z336570792 |  |  |  |  | 43 | ± | 6 | at | 387 | μM | 3 |  |
| AC149 | Z65773914 |  |  |  |  | 40 | ± | 4 | at | 393 | μM | 3 |  |
| AC212 | Z16668575 |  |  |  |  | 36 | ± | 4 | at | 41 | μM | 3 |  |
| AC060 | Z211829146 |  |  |  |  | 34 | ± | 12 | at | 410 | μM | 4 |  |
| AC215 | Z74370294 |  |  |  |  | 34 | ± | 4 | at | 15 | μM | 3 | Nonspecific ≥ 160 μM |
| AC166 | Z65674589 |  |  |  |  | 32 | ± | 4 | at | 37 | μM | 3 |  |
| AC008 | Z16668350 |  |  |  |  | 30 | ± | 6 | at | 39 | μM | 4 |  |
| AC175 | Z352403486 |  |  |  |  | 28 | ± | 7 | at | 407 | μM | 2 |  |
| AC055 | Z1132731573 |  |  |  |  | 25 | ± | 8 | at | 375 | μM | 3 |  |
| AC163 | Z13601968 |  |  |  |  | 24 | ± | 3 | at | 40 | μM | 2 |  |
| AC052 | Z1538134527 |  |  |  |  | 24 | ± | 4 | at | 395 | μM | 3 |  |
| AC081 | Z336571144 |  |  |  |  | 23 | ± | 8 | at | 366 | μM | 2 |  |
| AC054 | Z415105298 | - |  |  |  |  |  |  |  |  |  |  | No activity |
| AC056 | Z1427175725 | - |  |  |  |  |  |  |  |  |  |  | No activity |
| AC057 | Z1132789377 | - |  |  |  |  |  |  |  |  |  |  | No activity |
| AC064 | Z1689440691 | - |  |  |  |  |  |  |  |  |  |  | No activity |
| AC065 | Z954462332 | - |  |  |  |  |  |  |  |  |  |  | No activity |
| AC066 | Z448878758 | - |  |  |  |  |  |  |  |  |  |  | No activity |
| AC067 | Z448873790 | - |  |  |  |  |  |  |  |  |  |  | No activity |
| AC069 | Z434244550 | - |  |  |  |  |  |  |  |  |  |  | No activity |
| AC074 | Z212162276 | - |  |  |  |  |  |  |  |  |  |  | No activity |
| AC094 | Z54635424 | - |  |  |  |  |  |  |  |  |  |  | No activity |
| AC124 | Z1095441172 | - |  |  |  |  |  |  |  |  |  |  | No activity |
| AC125 | Z50923326 | - |  |  |  |  |  |  |  |  |  |  | No activity |
| AC126 | Z14472588 | - |  |  |  |  |  |  |  |  |  |  | No activity |
| AC145 | Z65773905 | - |  |  |  |  |  |  |  |  |  |  | No activity |
| AC147 | Z65845211 | - |  |  |  |  |  |  |  |  |  |  | No activity |
| AC160 | Z16336648 | - |  |  |  |  |  |  |  |  |  |  | No activity |
| AC164 | Z65682001 | - |  |  |  |  |  |  |  |  |  |  | No activity |
| AC170 | Z838953070 | - |  |  |  |  |  |  |  |  |  |  | No activity |
| AC171 | Z118607806 | - |  |  |  |  |  |  |  |  |  |  | No activity |
| AC172 | Z228758604 | - |  |  |  |  |  |  |  |  |  |  | No activity |
| AC174 | Z352403582 | - |  |  |  |  |  |  |  |  |  |  | No activity |
| AC176 | Z1011764818 | - |  |  |  |  |  |  |  |  |  |  | No activity |
| AC188 | Z255123788 | - |  |  |  |  |  |  |  |  |  |  | No activity |
| AC226 | Z14159896 | - |  |  |  |  |  |  |  |  |  |  | No activity |
| AC139 | Z14235133 | Nonspecific |  |  |  |  |  |  |  |  |  |  | Nonspecific |
| AC152 | Z65772333 | Nonspecific |  |  |  |  |  |  |  |  |  |  | Nonspecific |
| AC153 | Z66103417 | Nonspecific |  |  |  |  |  |  |  |  |  |  | Nonspecific |
| AC199 | Z16668565 | Nonspecific |  |  |  |  |  |  |  |  |  |  | Nonspecific |
| AC207 | Z18052577 | Nonspecific |  |  |  |  |  |  |  |  |  |  | Nonspecific |

**References**

1. Shehata, M. A. *et al.* Novel agonist bioisosteres and common structure-activity relationships for the orphan G protein-coupled receptor GPR139. *Sci. Rep.* **6**, 36681, doi:10.1038/srep36681 (2016).
